# Independent Origin of *Plasmodium falciparum* Antifolate Super-Resistance, Uganda, Tanzania, and Ethiopia

**DOI:** 10.3201/eid2008.131897

**Published:** 2014-08

**Authors:** Michael Alifrangis, Sidsel Nag, Mette L. Schousboe, Deus Ishengoma, John Lusingu, Hirva Pota, Reginald A. Kavishe, Richard Pearce, Rosalynn Ord, Caroline Lynch, Seyoum Dejene, Jonathan Cox, John Rwakimari, Daniel T.R. Minja, Martha M. Lemnge, Cally Roper

**Affiliations:** Centre for Medical Parasitology at Department of International Health, Immunology, and Microbiology, Faculty of Health and Medical Science, University of Copenhagen and at Department of Infectious Diseases, Copenhagen University Hospital (Rigshospitalet),; Copenhagen, Denmark (M. Alifrangis, S. Nag, M.L. Schousboe);; Tanga Research Centre, Tanzania (D. Ishengoma, J. Lusingu, D.T.R. Minja, M.M. Lemnge);; London School of Hygiene and Tropical Medicine, London, UK (H. Pota, R. Pearce, R. Ord, C. Lynch, J. Cox, C. Roper);; Kilimanjaro Christian Medical University College, Moshi, Tanzania (R.A. Kavishe);; Kilimanjaro Clinical Research Institute, Moshi (R.A. Kavishe);; Médécins Sans Frontières, Humera, Ethiopia (S. Dejene);; Ministry of Health of Uganda, Kampala, Uganda (J. Rwakimari)

**Keywords:** Dihydrofolate reductase, Pfdhfr, dihydropteroate synthetase, Pfdhps, 581, eastern Africa, intermittent preventive treatment in pregnant women, malaria, microsatellite, Plasmodium falciparum, resistance, single-nucleotide polymorphism, sulfadoxine-pyrimethamine, malaria, parasites, vector-borne infections, Uganda, Tanzania, Ethiopia

## Abstract

Super-resistant *Plasmodium falciparum* threatens the effectiveness of sulfadoxine–pyrimethamine in intermittent preventive treatment for malaria during pregnancy. It is characterized by the A581G *Pfdhps* mutation on a background of the double-mutant *Pfdhps* and the triple-mutant *Pfdhfr*. Using samples collected during 2004–2008, we investigated the evolutionary origin of the A581G mutation by characterizing microsatellite diversity flanking *Pfdhps* triple-mutant (437G+540E+581G) alleles from 3 locations in eastern Africa and comparing it with double-mutant (437G+540E) alleles from the same area. In Ethiopia, both alleles derived from 1 lineage that was distinct from those in Uganda and Tanzania. Uganda and Tanzania triple mutants derived from the previously characterized southeastern Africa double-mutant lineage. The A581G mutation has occurred multiple times on local *Pfdhps* double-mutant backgrounds; however, a novel microsatellite allele incorporated into the Tanzania lineage since 2004 illustrates the local expansion of emergent triple-mutant lineages.

## Abstract 2

Comprehensive surveillance is needed to support sulfadoxine–pyrimethamine intermittent preventive treatment in pregnant women.

Controlling and reducing malaria requires a combination of vector control measures and administration of antimalarial drugs as prophylaxis or treatment (*1*). The widespread use of antimalarial drugs has resulted in the emergence of resistant *Plasmodium falciparum*, recurrently exposing persons in malaria-endemic regions to an unacceptably high risk for treatment failures (*2*).

Highly chloroquine-resistant parasites spread from Asia in the 1960s and led to devastating rates of malaria-related death in Africa starting in the late 1980s, gradually forcing affected countries to replace chloroquine with sulfadoxine–pyrimethamine (SP) (*3*–*5*). The effectiveness of SP did not last long. In fact, retrospective analysis indicated that pyrimethamine-resistant parasites were present in sub-Saharan Africa before SP was implemented as first-line treatment, probably because pyrimethamine as monotherapy had been used in Asia during the 1960’s and 1970’s (*6*–*8*). Resistance to sulfadoxine also soon emerged (*9*), and the combination of pyrimethamine- and sulfadoxine-resistant parasites led to severe and widespread SP treatment failure (*10*–*12*). As a consequence, affected countries were once again forced to change their drug policies (*13*) and have now adopted artemisinin-based combination therapies as first-line treatment for uncomplicated malaria. Yet, SP is still recommended for use as intermittent preventive treatment in pregnant women (SP-IPTp) and infants (SP-IPTi) (*14*,*15*). Also, seasonal malaria chemoprevention applies SP in combination with amodiaquine (*16*). Use of SP for prevention in many countries of sub-Saharan Africa, where clinical failure after SP treatment has been reported, underscores the need for effective surveillance of its protective efficacy and for monitoring of the development and spread of SP resistance in *P. falciparum* populations.

The molecular basis of SP resistance is a combination of single-nucleotide polymorphisms (SNPs) in 2 distinct genes coding for the target enzymes of SP. The enzymes dihydrofolate reductase (DHFR) and dihydropteroate synthetase (DHPS) are targeted by pyrimethamine and sulfadoxine, respectively (*17*). High-level pyrimethamine resistance is generally encoded by 3 mutations in the *Pfdhfr* gene, coding for substitutions: N51I, C59R, and S108N (*18*); the molecular basis of sulfadoxine resistance is caused by substitutions S/A436F, A437G, K540E, A581G, and A613S/T in a variety of combinations in DHPS (*19*).

The most prevalent genotype in eastern Africa is a combination of the *Pfdhfr* triple mutant (51I, 59R, and 108N, denoted as IRN) combined with the *Pfdhps* double mutant (S436, 437G, 540E, A581, and A613, denoted as SGEAA). Together, this combination of SNPs is referred to as the “quintuple” mutant *Pfdhfr*/*Pfdhps* genotype and is associated with high risk for SP treatment failure (*17*) and results in limited protective value of SP-IPTi (*20*). Accordingly, the World Health Organization (WHO) recommends that SP-IPTi should be implemented only when the prevalence of the K540E mutation (and thus the quintuple mutant) is <50% (*14*).

More recently, an alanine to glycine mutation at codon position 581 in *Pfdhps* has emerged that, in combination with the *Pfdhfr* triple-mutant allele IRN, was shown to confer higher level resistance (*21*). This combination, referred to as the “sextuple *Pfdhfr*/*Pfdhps* mutant genotype” or the “super-resistant genotype” (*22*), is associated with reduced SP-IPTp efficacy by 1) a reduction in the protection period of SP-IPTp from 4 weeks to 2 weeks (*23*); 2) increased parasitemia attributed to competitive facilitation (*23*); 3) increased risk for severe malaria in the offspring (*24*); and 4) low birthweight in newborns from mothers undergoing SP-IPTp in Tanzania (*25*). Consequently, WHO recommendations concerning the use of SP-IPTp base the threshold on 2 mutations: SP-IPTp should be discontinued if the prevalence of the K540E mutation is >95% and the A581G mutation is >10% (*20*). No threshold in the prevalence of molecular markers of resistance has been set with regard to seasonal malaria chemoprevention (*15*,*16*).

Maps collating all published data from molecular surveillance of *Pfdhfr* and *Pfdhps* mutations (*22*) indicate 3 main foci of super-resistant parasites: 1 in northern Tanzania (*26*); a second in southwestern Uganda, Rwanda, and bordering areas of Democratic Republic of Congo (*27*–*29*); and a third in western Kenya (*30*). Prevalence of A581G also is high in Ethiopia and northern Sudan, where it again occurs as the *Pfdhps* triple-mutant allele SGEGA but in combination with a *Pfdhfr* double-mutant allele 51I-108N.

Assessments of microsatellite variation linked to *Pfdhps* have shown that limited microsatellite diversity flanking the SGEAA double mutants compared with the SAKAA wild types. Two SGEAA lineages were discovered in eastern Africa: 1 prevailing in northeastern Africa (Ethiopia and Sudan) and the other throughout southeastern Africa. Both lineages derived from independent ancestry (*10*). Here we apply the same approach, using the same microsatellite loci, to determine the ancestry and possible relationship between the double SGEAA and triple SGEGA alleles in Ethiopia, Uganda, and Tanzania. By focusing on microsatellite variation linked to *Pfdhps*, we can explore whether the emergence of the SP-IPT-threatening SGEGA triple mutants in Ethiopia, Uganda, and Tanzania derive from local SGEAA alleles or are being imported.

## Materials and Methods

### Study Sites

Samples for the study were collected during 2004–2008. Study sites were in Uganda (2 sites), Tanzania (3 sites), and Ethiopia (1 site) (Table).

**Table:** Study sites and number of samples genotyped for super-resistant *Plasmodium falciparum*, eastern Africa

Study site.	SGEAA			SGEGA		Total haplotype diversity
	No. samples	Haplotype diversity		No. samples	Haplotype diversity	
Ethiopia, Humera, Tigray	35	4		41	1	4
Uganda						
Bufundi, Kabale	24	5		14	3	7
Kebisoni, Rukungiri	27	5		14	3	7
Tanzania						
Hale, Tanga	21	6		15	3	8
Korogwe, Tanga	64	21		19	3	22
Magoda, Tanga	7	3		15	6	6

*By country, district, village. Samples from Ethiopia are from 2004; samples from Uganda are from 2005; and samples from Tanzania are from 2004 (Korogwe only), 2006 (Korogwe and Hale), 2007 (Korogwe only) and 2008 (Magoda only).

### Sample Collection

#### Bufundi and Rikungiri, Uganda

Uganda implemented SP-IPTp in 2000 and has not implemented SP-IPTi or seasonal malaria chemoprevention. Finger-prick blood-spot samples were obtained from symptomatic patients of all ages after *P. falciparum* infection was confirmed by a Paracheck rapid test (Orchid Biomedical Systems, Chennai, India) during May–December 2005 at reference health facilities in Bufundi (Kabale District) (38 samples) and Kebisoni (Rukungiri District) (41 samples). Blood spots were air dried on Whatman no. 3 filter paper (VWR–Bie & Berntsen, Herlev, Denmark), sealed in plastic bags with a desiccant, and stored at room temperature for molecular genotyping (*27*). The Uganda National Council for Science and Technology (UNSCT HS 35) and the ethics committee of the London School of Hygiene and Tropical Medicine (London, UK) gave scientific and ethical permission. Consent was obtained from all persons or their guardians before sample collection.

#### Hale, Korogwe, and Magoda, Tanzania

Tanzania implemented SP-IPTp in 2001 and has not implemented SP-IPTi or seasonal malaria chemoprevention. Samples were obtained from 3 different settings in Tanga region. From Hale (36 samples), finger-prick blood-spot samples were taken from symptomatic children 6–59 months of age who attended Hale Health Centre during July–August 2006 as previously described (*21*). The study protocol was approved by the Ethics Review Committees of the National Institute for Medical Research, Tanzania, and the London School of Hygiene and Tropical Medicine and was registered as a clinical trial with the National Institutes of Health (http://www.clinicaltrials.gov, identifier NCT00361114). From Korogwe (83 samples), finger-prick or venous blood samples were obtained on filter paper from children and adolescents <20 years of age from Mkokola and Kwamasimba villages. Samples were collected in March 2004, May 2006, and May 2007, as described (*26*). The Medical Research Coordinating Committee of the National Institute for Medical Research and Ministry of Health, Tanzania, granted ethical clearance for the study. All participants or their parents or guardians provided informed consent. Samples from Magoda villages (22 samples) were collected from children <5 years of age in June 2008 as part of a cross-sectional assessment of malaria prevalence (*31*).

#### Humera, Ethiopia

Ethiopia has adopted neither of the WHO recommendations regarding use of SP as prophylaxis. Samples were collected from patients of all ages who attended Kahsay Abera Hospital in Humera during January–April 2004 and who had symptomatic uncomplicated malaria (*10*). The patients were enrolled in an in vivo efficacy trial, comparing artemether–lumefantrine therapy with SP therapy, which was conducted by staff of Kahsay Abera Hospital and the Mekele Regional Health Bureau. Finger-prick blood-spot samples were taken from patients before treatment after they gave written informed consent to participate in the study, and genetic analysis was conducted in support of the drug efficacy evaluation. The Ethical Clearance Committee of the Tigray Health Research Council and the external Ethics Review Board used by Médecins sans Frontières gave ethical permissions for the study.

### Genotyping

Sample collection at the different study sites was not standardized because the samples derived from independent studies. However, all samples consisted of finger-prick blood spots stored on filter paper, and parasite DNA was extracted by using the Chelex method (*32*).

Point mutations in samples from Hale, Tanzania, were determined by direct sequencing (*21*). Sequencing was performed by using the ABI-3730 automatic sequencer (Applied Biosystems, Foster City, CA, USA), and samples were analyzed with Applied Biosystems BigDye V. 3.1 (Applied Biosystems).

For all other samples, the polymorphic region of *Pfdhps* was PCR-amplified before sequence-specific oligonucleotide probing (SSOP) for mutations at codons 436, 437, 540, 581, and 613 by using primers and PCR conditions described elsewhere (*33*). SSOP-genotyping of samples from Uganda and Ethiopia was conducted according to an SSOP–dot-blot method (*10*); genotyping of samples from Korogwe and Magoda in Tanzania was conducted according to an SSOP-ELISA method (*33*).

Only samples containing the *Pfdhps* SGEAA or SGEGA alleles were included for further analysis; other alleles, such as wild-type or single-mutant alleles, were excluded. In general, only a single sequence was detected at every codon, but if the sequence analysis detected a mixture, these samples were handled as mixed infections. Mixed infections, in turn, were further analyzed only if 1 allele was substantially in the majority (i.e., a 2:1 signal ratio between the dominant genotype and the minor genotype) and a majority SNP could be confidently determined at all codon positions (*33*).

### Microsatellite Analysis

Analysis was performed on 3 *Pfdhps-*linked microsatellites located 0.8 kb (marker [m.] 0.8), 4.3 kb (m. 4.3), and 7.7 kb (m. 7.7) downstream of the coding position 437 of *Pfdhps*, located on chromosome 8 (*34*). Microsatellites were amplified by seminested PCR as described previously (*34*), and products were run with GeneScan-500 LIZ Size Standards (Applied Biosystems) in an ABI 3730 DNA analyzer (Applied Biosystems) and analyzed by using Genemapper software (Applied Biosystems). If >1 microsatellite allele was detected in any given sample, the peak height ratio was used to determine the majority allele for that locus. If the major allele did not have a peak height of at least double the height of the minor allele, the sample was excluded from further analysis.

Microsatellite haplotypes were constructed by combining alleles detected in each of the 3 microsatellite loci. Samples with missing data were not included.

## Results

A total of 300 samples with either *Pfdhps* double-mutant (SGEAA) or triple-mutant (SGEGA) alleles were subjected to microsatellite analysis, and 277 (92.3%) of these gave conclusive results. Microsatellite haplotypes associated with the *Pfdhps* double- and triple-mutant alleles are listed in full (Technical Appendix Table), where the haplotypes are ranked hierarchically according to allele size, first at the 0.8-kb locus, then at 4.3-kb locus, and finally at the 7.7-kb locus, and each unique haplotype was assigned a number.

### Diversity of Microsatellite Composition among SGEAA Samples

SGEAA alleles from Ethiopia were associated with 4 different microsatellite haplotypes (Figure, panel A; Table). Haplotype 4 (notation 121–114–98, refers to fragment size 121 bp at the 0.8-kb locus, 114 bp at the 4.3-kb locus, and 98 bp at the 7.7-kb locus) predominated and was found in 30 (85.7%) of the 35 SGEAA alleles sampled. Of the remaining 3 microsatellite haplotypes, haplotypes 7 and 21 were each found twice; 11 was found once. Haplotype 11 (131–104–107) was most common in the samples from Uganda and Tanzania. No samples from Tanzania or Uganda were Ethiopia haplotype 4. The Uganda SGEAA alleles were associated with 8 different microsatellite haplotypes (Figure, panel A; online Technical Appendix Table, 5 haplotypes found at each site); the most common haplotype, haplotype 11 (131–104–107), was found in 82.4%, (42/51) of samples. The 92 SGEAA samples collected from 3 study sites in Tanzania exhibited 24 different microsatellite haplotypes (Figure, panel A; online Technical Appendix Table). Korogwe exhibited the greatest diversity by having 21 haplotypes among 64 SGEAA sample. As in Uganda, most of the Tanzania SGEAA alleles were associated with haplotype 11 (53 [57.6%]). Among the remaining 39 samples were 23 alternative but related haplotypes.

**Figure:**
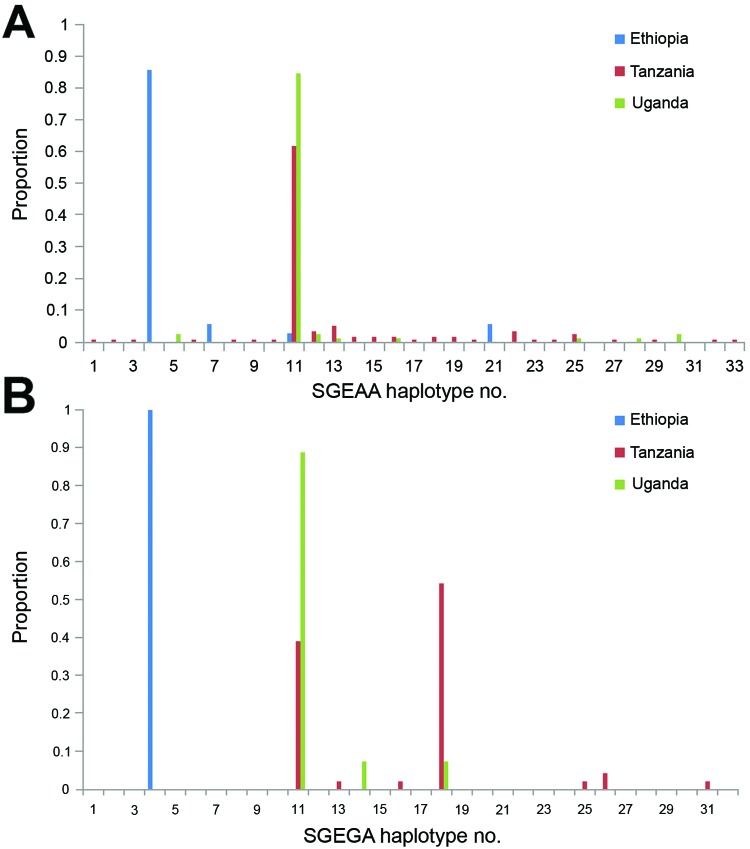
Proportion of microsatellite haplotypes linked to SGEAA and SGEGA, eastern Africa. Microsatellite haplotypes associated with the *Pfdhps* double-mutant allele SGEAA (A) and *Pfdhps* triple-mutant allele SGEGA (B) in Ethiopia, Tanzania, and Uganda. Haplotype numbering (x-axis) refers to a unique combination of microsatellite allele sizes at the 3 loci linked to *dhps* (specific microsatellite allele combinations are listed in the Technical Appendix Table, Proportion (y-axis) is the number of alleles associated with each microsatellite haplotype expressed as a proportion of the total number of alleles sampled in each country for which the associated microsatellite haplotype could be determined.

### Diversity of Microsatellite Composition among SGEGA Samples

Of 41 SGEGA samples from Ethiopia, only 1 microsatellite haplotype was present: haplotype 4 (121–114–98) (Figure, panel B; Technical Appendix Table). The 28 SGEGA samples from Uganda were associated with 3 microsatellite haplotypes; all 3 combinations were represented in the 2 sites (Figure, panel B; Appendix Table). Of these SGEGA samples, 24 (85.7%) were haplotype 11 (131–104–107). The 2 less common haplotypes, haplotypes 18 (131–104–125) and 14 (131–104–113), are evidently related to haplotype 11, differing by 1 allele at the 7.7-kb locus. Of 49 SGEGA samples collected from Tanzania, we found 7 microsatellite haplotypes (Figure, panel B; Technical Appendix Table). This finding indicates less diversity than was associated with SGEAA alleles: Hale (3 haplotypes), Korogwe (3 haplotypes), and Magoda (6 haplotypes). Of the Tanzania SGEGA samples, 18 (36.7%) were haplotype 11, the same haplotype common among Tanzania and Uganda SGEAA samples and among Uganda SGEGA samples. Of the remaining 28 SGEGA samples from Tanzania, 24 (49.0% of total Tanzania SGEGA samples) were haplotype 18 (131–104–125), a haplotype found only twice in association with SGEAA samples from Tanzania. This haplotype was found twice among Uganda SGEGA samples and never among Uganda SGEAA samples.

## Discussion

Although SP is no longer recommended as first-line treatment for *P. falciparum* infection, it is widely recommended for prevention and possibly still available over the counter for self-treatment. Presumably, therefore, SP has continued to exert selective pressure on already resistant parasites, which might explain the continuing emergence of the triple-mutant *Pfdhps* allele (SGEGA), which is currently being described for certain regions of eastern Africa. An increased prevalence of the A581G mutation has been well documented in eastern Africa in recent years; it increased from 12% in 2003 to 56% in 2007 at study sites in Korogwe, Tanzania (*26*). In Kabale and Rukungiri, Uganda, samples from 2005 showed a high prevalence of the A581G mutation at 45% and 46%, respectively (*27*); studies during 2005–2006 in Rukara and Mahesha, Rwanda, observed prevalences of 60% and 29%, respectively (*28*). A study in 2010 in Huye District, Southern Province, Rwanda, reported a prevalence of 63% (*35*). In eastern Sudan, a study found an increase in the prevalence of the A581G mutation from 14% in 2003 to 34% in 2012 (*36*). In Kenya, Kisumu, a study showed an increase in the A581G mutation from 0% in 1999–2000 to 85% in 2003–2005 (*30*). More recently, in Nyanza Province, western Kenya, the prevalence of the A581G increased from 0% to 5.3% from 2008–2009 (*37*).

In this study, we investigated the origins of triple-mutant *Pfdhps* alleles by analyzing the microsatellite diversity flanking *Pfdhps*. We sampled both SGEAA double mutants and SGEGA triple mutants in 3 populations at the key moment: when the SGEGA triple mutant had emerged but had not yet replaced the SGEAA double mutant. At this time, double- and triple-mutant alleles were present in similar numbers in the areas, but SP-sensitive alleles were very rare.

In Ethiopia, both SGEAA and SGEGA alleles were associated with haplotype 4 (121–114–98), indicating a shared ancestry that has evolved independently from SGEAA and SGEGA alleles from Uganda and Tanzania. The SGEAA in these 2 countries were associated with lineage 11 (131–104–107), the same microsatellite haplotype previously shown to be associated with the double-mutant alleles throughout Tanzania, Kenya, Uganda, Mozambique, and Zambia (*10*).

In Ethiopia and Uganda, we found evidence that the most prevalent SGEAA haplotype locally had given rise to SGEGA haplotypes in the same area; the most common SGEAA haplotype was also the most common SGEGA haplotype in both countries (haplotypes 4 and 11, respectively). However, in Tanzania, the microsatellite haplotype most commonly associated with SGEGA (haplotype 18 [131–104–125], 25/49 samples) was not identical to that most commonly associated with SGEAA (haplotype 11 [131–104–107]), because only 2 SGEAA samples were haplotype 18. This finding leads us to speculate that the A581G mutation has emerged on at least 2 occasions in Tanzania.

We found that the microsatellite diversity associated with both SGEAA and SGEGA haplotypes in Ethiopia samples was less than in the SGEAA and SGEGA samples from sites in Uganda and Tanzania. The high level of homozygosity among microsatellite haplotypes in Ethiopia might be due to a high degree of selective pressure, which in turn might be assisted by population bottlenecks brought about by a narrow malaria transmission season and limited exchange of parasites, with neighboring regions resulting from limited migration. The Ethiopia samples originate from the Tigray District near the Eritrean border. Despite some migration of refugees from Eritrea to Ethiopia, a substantial spread to and from the Tigray District in Ethiopia during the years before sample collection is doubtful because of the continued presence of forces at the border during the cease fire succeeding the Eritrean–Ethiopian war initiated in 2000. Double- and triple-mutant alleles from northeastern and eastern Sudan also are associated with haplotype 4 (121–114–98) (*10*) and represent greater diversity than what we present from Ethiopia, which supports the view that the parasite populations in these 2 countries are linked (*10*,*38*).

Tanzania is a capital of trade and emigration for sub-Saharan Africa. The Tanzam highway (running from Tanzania through Zambia) is one of the most trafficked roads on the African continent, and higher diversity and sharing of common microsatellite haplotypes among the Ugandan and Tanzanian populations were therefore expected. A recent publication about the correlation between human population movement and malaria movement in Uganda, Tanzania, and Kenya (*39*) illustrates that the major human population movement and malaria movement in Tanzania originates from central Dodoma and directs northward and westward. In this regard, an early selection of a haplotype in the Tanga region (northeast) compared with other areas of Tanzania, can be speculated to be plausible because of the larger levels of parasite migration to northern and western parts of the country, diluting to some extent the newly selected haplotypes in these areas.

In conclusion, we provide evidence that the A581G mutation can arise on various SGEAA ancestral backgrounds, of which we have shown 3 different cases (haplotypes 4, 11, and 18), from areas previously known to represent 2 distinct parasite lineages. Our microsatellite analysis is consistent with reports that the SGEGA triple-mutant alleles are undergoing rapid expansion, and we found evidence of spread of the Tanzania SGEGA haplotype (haplotype 18) as far as southwestern Uganda, which illustrates the potential for dispersal of super-resistant *P. falciparum* malaria throughout the region. Given the rate of increase and the ability of double-mutant allele lineages to acquire the super resistance–conferring A581G mutation independently, it is vital for the continuing effectiveness of prophylaxis with SP that more comprehensive surveillance for the A581G mutation be used to track emerging super-resistant malaria in Africa.

Technical Appendix Microsatellite haplotypes and their geographic occurrence and association with double- and triple-mutant *Plasmodium falciparum* *dhps* alleles.
