# Supplementary material for: Independent Origin of Plasmodium falciparum Antifolate Super-Resistance, Uganda, Tanzania, and Ethiopia
Source: Emerg Infect Dis. 2014 Aug;20(8):1280–6. doi: 10.3201/eid2008.131897 (PMC4111169; doi:10.3201/eid2008.131897)
Supplement: Technical Appendix — Microsatellite haplotypes and their geographic occurrence and association with double- and triple-mutant Plasmodium falciparum dhps alleles. [file 13-1897-Techapp-s1.pdf]

Article DOI: http://dx.doi.org/10.3201/eid2008.131897

Super-Resistant *Plasmodium falciparum*, Uganda, Tanzania, and Ethiopia

Technical Appendix

Technical Appendix Table. Microsatellite haplotypes and their geographic occurrence and association with double and triple mutant *Plasmodium falciparum dhps* alleles*

Haplotype	Locus				Tanzania			Uganda			
no.	0.8	4.3	7.7	Ethiopia	Hale	Korogwe	Magoda	Kabale	Rikungiri	SGEAA	SGEGA
1	117	104	107			1				1	
2	117	108	107			1				1	
3	121	106	107			1				1	
4	121	114	98	71						30	41
5	123	108	109						2	2	
6	123	108	119			1				1	
7	123	114	98	2						2	
8	125	110	119			1				1	
9	129	98	119			1				1	
10	131	100	107			1				1	
11	131	104	107	1	23	38	10	32	34	96	42
12	131	104	109			3	1	1		5	
13	131	104	111			5	1		1	6	1
14	131	104	113			2		1	1	2	2
15	131	104	115		1	1				2	
16	131	104	117		1		1	1		2	1
17	131	104	121				1			1	
18	131	104	125		6	14	6	1	1	2	26
19	131	104	127			2				2	
20	131	104	129		1					1	
21	131	106	119	2						2	
22	131	110	113		2	2				4	
23	131	110	119			1				1	
24	131	114	111			1				1	
25	133	104	107		1	2			1	3	1
26	133	104	125			2					2
27	133	104	129		1					1	
28	133	104	136					1		1	
29	136	103	115			1				1	
30	136	104	107					1	1	2	
31	136	104	125				1				1
32	136	110	117			1				1	
33	138	110	113			1				1	

*For each unique haplotype, the fragment size in bp at each of the 3 linked microsatellite loci is shown. Blank cells indicate that the haplotypes were not found in the respective countries.
